# In vivo magnetic resonance imaging evidence of olfactory bulbs changes in a newborn with congenital Citomegalovirus: a case report

**DOI:** 10.1186/s13052-021-01170-w

**Published:** 2021-11-16

**Authors:** Andrea Bianchi, Caterina Coviello, Valentina Leonardi, Michele Luzzati, Stefano Chiti, Daniele Ermini, Vittorio Miele, Enrico Fainardi, Carlo Dani, Elisa Scola

**Affiliations:** 1 Department of Neuroradiology, Careggi University Hospital, Largo Piero Palagi 1, Florence, Italy; 2 Division of Neonatology, Careggi University Hospital, Largo Piero Palagi 1, Florence, Italy; 3 Department Health Professions, U.O. Research and Development, Careggi University Hospital, Largo Piero Palagi 1, Florence, Italy; 4 Department of Emergency Radiology, Careggi University Hospital, Largo Brambilla 3, 50134 Florence, Italy; 5 Department of Scienze Biomediche, Sperimentali E Cliniche, Neuroradiology, University of Florence, Viale Morgagni, 50 Florence, Italy; 6 Department of Neurosciences, Psychology, Drug Research and Child Health, Careggi University Hospital, Largo Piero Palagi 1, Florence, Italy

**Keywords:** Congenital Citomegalovirus, Newborn, Magnetic resonance imaging, Olfactory bulbs, Case report

## Abstract

**Background:**

Citomegalovirus (CMV) infects approximately 1% of live newborns. About 10% of the infants affected by congenital CMV infection are symptomatic at birth and up to 60% of these infants will develop permanent neurological disabilities. Depending on gestational age (GA) at the time of infection, the involvement of central nervous system (CNS) can lead to malformations of cortical development, calcifications, periventricular white matter lesions and cysts, ventriculomegaly and cerebellar hypoplasia.

**Case presentation:**

We report the MRI findings in a Caucasian female born at 32 weeks of post-menstrual age with post-birth diagnosis of congenital CMV infection showing an unusual and peculiar marked T2 hyperintensity of the inner part of olfactory bulbs in addition to the CMV related diffuse brain involvement. Despite the known extensively described fetal and neonatal Magnetic Resonance Imaging (MRI) findings in CMV infected fetuses and newborns, any in vivo MRI depiction of olfactory system damage have never been reported so far. Nevertheless, in murine studies CMV is known to infect the placenta during pregnancy showing particular tropism for neural stem cells of the olfactory system and previous neuropathologic study on CMV infected human fetal brains from 23 to 28 weeks of GA reported damage in the olfactory bulbs (OB) consisting in disseminated cytomegalic cells, inflammation, necrosis and neuronal and radial glial cell loss. Therefore, we assume an OB involvement and damage in congenital CMV infection.

**Conclusion:**

To our knowledge this is the first in vivo MRI evidence of OB damage in a newborn with congenital CMV infection that may give new insights on CMV infection.

## Background

Citomegalovirus (CMV) infects approximately 1% of live newborns. About 10% of the infants affected by congenital CMV infection are symptomatic at birth and up to 60% of these infants will develop permanent neurological disabilities [1, 2]*.* During pregnancy CMV may infect the placenta and may cause fetal growth retardation, severe brain malformations and sensorineural impairment [3]. Depending on gestational age (GA) at the time of infection, the involvement of central nervous system (CNS) can lead to malformations of cortical development, calcifications, periventricular white matter lesions and cysts, ventriculomegaly and cerebellar hypoplasia [4]. Fetal and neonatal Magnetic Resonance Imaging (MRI) findings in CMV infected fetuses and newborns have already been extensively described [5, 6] and CMV is known to show particular tropism for neural stem cells of the olfactory system [7]. Despite this, any in vivo MRI depiction of olfactory system damage have never been reported so far.

## Case presentation

A Caucasian female was born at 32 weeks of GA to a 36-year-old woman via emergency cesarean section because of alterations in the fluximetric indices. TORCH screening performed during pregnancy resulted negative. Intrauterin growth retardation was evidenced since 26 weeks of GA. The APGAR scores were 7 and 8 at 1 and 5 min, respectively. Birth weight was 1210 g (6 th percentile), length was 39 cm (13 th percentile), and head circumference was 26 cm (1 th percentile). She was the second baby born to nonconsanguineous parents. The infant was admitted to the Neonatal Intensive Care Unit (NICU) because of respiratory distress syndrome and received nasal CPAP for 7 days. Physical examination at birth revealed generalized petechial rash and splenomegaly, and a complete blood count evidenced thrombocytopenia (22,000/mm3). Leukopenia (white blood cell count 4900/mm3) and anemia with 10.4 g/dL of hemoglobin were observed since the fifth day of life (DOL). The diagnosis of congenital CMV infection was proven by the detection, by polymerase chain reaction, of CMV DNA on a urine sample (32,000 copies/mL) and on blood sample (71,800 copies/ml). *Cephalo-rachidian fluid* also tested positive (390 copies/ml). Serological results revealed positive IgM and IgG antibodies anti-CMV. The mother did not performe CMV serology during pregnancy but preconceptional maternal immunity showed positive CMV-IgG and negative CMV-IgM. Thus, the infection was caused by maternal re-infection. Intravenous ganciclovir was started on sixth DOL and was switched to oral valganciclovir as soon as feeding tolerance was achieved (16 mg/kg twice a day). After the first week the infant developed cholestasis without signs of hepatitis. During the recovery the infant received numerous platelet and red blood cells transfusions. Granulocyte colony-stimulating factor (G-CSF) was administrated for the neutropenia. Since birth, clinical neurological evaluation revealed axial hypotonia and irritability alternating to lethargy. Serial cranial ultrasound showed periventricular cysts, lenticulostriate vasculopathy cysts and bilateral germinal matrix haemorrhage. MRI scan of the brain was acquired at 37 weeks GA. MRI scan was performed on a 1.5 Tesla scanner (Siemens Magnetom Aera Erlangen Germany, release VE11C). Axial and coronal T2-weighted images, volumetric isotropic sagittal T1 weighted Magnetization Prepared Rapid Acquisition Gradient Echo (MPRAGE) image, axial Diffusion Weighted Imaging Echo Planar Imaging Spin Echo (DWI EPI SE) image, volumetric axial Susceptibility Weighted Imaging (SWI) image, volumetric isotropic axial T2/T1 Weighted True Fast Imaging with Steady state free Procession (TrueFISP) image, were acquired.

**Fig. 1:**
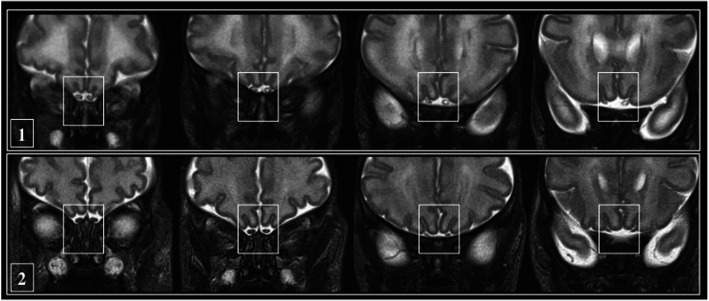
MRI coronal T2 weighted images: the OBs (squared white box) in the patient with CMV infection (row 1) showed abnormal T2 hyperintensity in the central portion in comparison with the physiological appearance (row 2) of OB of a newborn without CMV infection studied at the same corrected GA. CMV: Citomegalovirus, GA: gestational age; OB: olfactory bulb

**Fig. 2:**
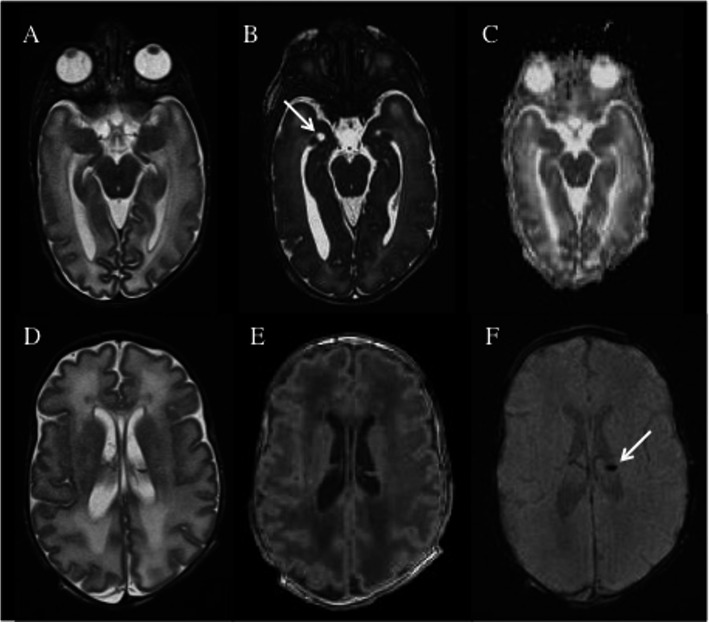
MR scan show an immature aspect of the gyration of the cerebral hemispheres associated with a reduced myelination (A and D, axial T2-weighted images; C, axial Diffusion Weighted Imaging DWI; E, volumetric axial-reformatted T1 weighted image). T2-weighted sections (A and D) revealed increased hyperintensity of the parieto-occipital and temporal white matter with cysts in the bilateral periventricular temporal region (white arrow in B, True Fast Imaging with steady state procession (TrueFISP) axial isotropic volumetric T2 weighted image) . Germinolytic cysts with hemosiderin deposit were depicted in the thalamocaudal notch region bilaterally (white arrow in F, axial Susceptibility Weighted Imaging (SWI))

The patient was sedated with Intranasal dexmedetomidine (2 mcg/kg) and continuously monitored for oxygen saturation and heart rate. MRI scan showed an immature aspect of the gyration of the cerebral hemispheres associated with a reduced myelination of the posterior arm of the internal capsule. T2-weighted sections revealed increased hyperintensity of the parieto-occipital and temporal white matter with cysts in the bilateral periventricular temporal region. Germinolytic cysts with hemosiderin deposit were depicted in the thalamocaudal notch region bilaterally. Interestingly, a market T2 hyperintensity of the inner part of olfactory bulbs was noticed (Figs. 1 and 2). Any abnormalities in the remaining cranial nerves weren’t found. She was given two CMV IgG transfusions without any improvement of thrombocytopenia, neutropenia and anaemia. Eye examination resulted negative for chorioretinitis and the hearing screen showed normal brain auditory evoked response. Due to long-lasting pancytopenia the infant was transferred to the haematological unit of the referral hospital on day thirty-eighth after birth. The parental informed consent for publication was obtained.

## Discussion and conclusions

Fetal and neonatal MRI findings in CMV infected fetuses and newborns have already been extensively described [5, 6]. Malformations of cortical development, calcifications, periventricular white matter lesions and cysts, ventriculomegaly and cerebellar hypoplasia occur according to GA at the time of infection [4]. Despite this, any in vivo MRI depiction of olfactory system damage has been never reported so far.

Nevertheless, previous neuropathologic study on CMV infected human fetal brains from 23 to 28 weeks of GA reported damage in the olfactory bulbs consisting in disseminated cytomegalic cells, inflammation, necrosis and neuronal and radial glial cell loss. Supporting this evidence, pronounced olfactory deficits were described in mouse model of CMV infection [8] occurring long before the auditory deficits. Additionally, studies on murine CMV showed that the placental CMV inoculation of embryos leads to olfactory bulbs infection. The virus enters via the apical cilia of olfactory sensory neurons (OSN) located in the nasal olfactory epithelium. The OSN project to olfactory bulbs (OB) where they form synapses with mitral/tufted cells whom axons directly connect with neurons of primary olfactory cortex. Additionally, CMV secondary spread systemically to blood through the myeloid cells that infiltrate the olfactory epithelium, become infected and then migrate in the superficial cervical lymph nodes [9]. In murine models OB infection was detected until 16 weeks after birth showing a longlasting persistence over time [8]. Interestingly the olfactory infection is a common, conserved route of mammalian herpesvirus entry to host [8, 10].

Olfaction develops antenatally before audition and vision. Nevertheless, the assessment of olfactory function is challenging as no specific tests on in newborns are available. However, the early detection of olfactory deficits might be relevant, given its function in fetuses and newborns in learning of maternal odors, guiding feeding and social behaviors and maintaining a strong parent–infant bound [11].

The radial glial cells surrounding the periventricular germinal epithelium and the progenitor cells within the germinal areas (the Ventricular Zone (VZ) and the Subventricular Zone (SVZ)) are of utmost importance for fetal brain development. Previous neuropathological studies in human fetal brains showed that the radial glial cells surrounding the periventricular germinal epithelium are the main cellular target of CMV infection. Additionally, the presence of CMV-infected cells in the SVZ and in the VZ, and in cortical plate and subplate too, was also depicted. It was shown that CMV infects cells exhibiting the phenotypic characteristics of neural stem cells/progenitors [7, 8]. These findings may explain the severe cellular loss in the VZ and SVZ occurring in CMV infected brain human fetus [7] associated to the impairment of brain development. Interestingly, a connection between the OB and the periventricular area have been suggested in human fetal brain. In support of this, pathological studies in fetal brain demonstrated the presence of a rostral migratory stream of neuroblasts coming from the SVZ and connecting the anterior horn of the lateral ventricle and the OB. Additionally, an extension of the lateral ventricle reaching the olfactory bulb and probably closes during fetal development, was described in human brains [12–14]. Therefore, in human fetal brain a connection between the periventricular area, where CMV infects cells of the VZ and SVZ, and the OB is probably present and may contribute to the spread of CMV infection within the fetal CNS.

The MRI in vivo involvement of OB in CMV infected newborns hasn’t been described so far. A previous study showed the pattern of physiological MRI appearance of OB from birth to adult age [15]: in newborns at 15 days of median age the OB are depictable as two hypointense oval structures with a less hypointense central areas in T2- weighted images. The central part was interpreted as an area of axons and synaptic networks connected to primary olfactory area and to ganglionic eminence still with immature myelination and rich in extracellular matrix; this area undergoes progressive myelination, similar to cerebral white matter, reduction of the extracellular matrix and is no more detectable in children older than 2 years of age. In the patient described the central portion of the OB showed noticeable abnormal T2 hyperintensity, while the surrounding peripheral nerve portion was spared (Fig. 1). It can be assumed that the central part is highly susceptible to damage due to its immaturity. As no specific tests for olfactory functions on newborns are available, MRI still remain a unique test for the assessment of central olfactory system in newborns. The lack of confirmation of olfactory dysfunction is the main limitation in the case presented and it may be argued that the appearance of OB described is due to immaturity. Nevertheless, Fig. 1 shows the physiological appearance of OB in a newborn without CMV infection and studied at the same corrected GA, where any severe signal alteration in OB is present, therefore it is unlikely that the OB MRI appearance would be due to immaturity. Interestingly, any changes weren’t observed in the other cranial nerves, in particular in the optic nerves that are a true neocerebral extension, such as the olfactory nerves, suggesting a specific involvement of OB by CMV infection.

## Conclusion

Despite several neuroimaging studies on fetuses and newborns with congenital CMV infection, this is the first in vivo evidence of olfactory bulbs damage in a newborn with congenital CMV infection. In the future it would be interesting to assess the OB damage of congenital CMV infection in retrospective and prospective MRI studies. These observations may give new insights on CMV infection, prevention and control.

## Data Availability

Any data or additional MRI images analyzed for this case report are available from the corresponding author on reasonable request.

## Abbreviations

CMV: Citomegalovirus
CNS: Central Nervous System
DOL: day of life
DWI: Diffusion Weighted Imaging
GA: Gestational Age
MRI: Magnetic Resonance Imaging
NICU: Neonatal Intensive Care Unit
OB: olfactory bulbs
OSN: olfactory sensory neurons
SVZ: Subventricular Zone
SWI: Susceptibility Weighted Imaging
TrueFISP: True Fast Imaging with steady state procession
VZ: Ventricular Zone
